# Impaired wound healing in type 1 diabetes is dependent on 5-lipoxygenase products

**DOI:** 10.1038/s41598-018-32589-7

**Published:** 2018-09-21

**Authors:** Theresa Ramalho, Luciano Filgueiras, Ildefonso Alves Silva-Jr, Ana Flavia Marçal Pessoa, Sonia Jancar

**Affiliations:** 1 Department of Immunology, Institute of Biomedical Sciences, University of São Paulo, São Paulo, Brazil; 2 Department of Cell and Developmental Biology, Institute of Biomedical Sciences, University of São Paulo, Sao Paulo, Brazil

## Abstract

Type 1 diabetes is associated with systemic low grade inflammation (LGI). We have previously shown that LGI in diabetic mice depends on systemic circulation of leukotriene (LTB_4_) which potentiates the *toll-like/*IL1β receptors response in macrophages. Impaired wound healing is an important co-morbidity in diabetes, and macrophages play a key role in this process. Here, we investigated the role of leukotrienes on monocytes and macrophages phenotype and in the impaired wound healing in diabetic mice. Type 1 diabetes was induced with streptozotocin in 129SvE wild-type (WT) and leukotrienes-deficient 5LO^−/−^ (5-lipoxygenase *knockout*) mice. In diabetics, the systemic levels of LTB4, TNF-α, IL-6, IL-10, IL-12 and IFNγ were increased as well as the frequency of pro-inflammatory monocytes (CD11b^+^Ly6C^high^Ly6G^−^) compared to healthy mice. In diabetic 5LO^−/−^ mice, these parameters were similar to those in healthy mice. Resident peritoneal macrophages from diabetic WT mice showed a classically activated M1-like phenotype (high *Nos2*, *Stat* and *Il12* expression, and nitrite levels). Macrophages from diabetic 5LO^−/−^ mice presented alternatively activated M2-macrophages markers (high *Arg1* and *Chi3l3* expression and arginase activity) and when stimulated with IL4, enhanced phosphorylated-STAT6. Cutaneous wound healing in diabetic WT mice was impaired, which correlated with the decreased frequency of M2-macrophages (CD45^+^F4/80^+^CD206^+^) in the lesions. In diabetic 5LO^−/−^ mice, the frequency of M2-macrophages in the wound was similar to that in healthy mice, suggesting that the impaired healing of diabetic mice depends on 5LO products. The inhibition of leukotrienes or antagonism of its receptors could be a therapeutic alternative for diabetic patients with impaired healing.

## Introduction

Type 1 diabetes (T1D) is a metabolic disorder characterized by chronic hyperglycemia and alterations in carbohydrate, lipid and protein metabolism. It develops because of an autoimmune response against pancreatic β-cells which leads to failed insulin production^1^. According to estimates, the incidence of type 1 diabetes is increasing by around 3–4% every year, particularly among children^2,3^.

Diabetic patients develop a chronic low grade inflammation (LGI) characterized by a chronic production of pro-inflammatory cytokines, such as TNF-α, IL-1β and IL-6^4,5^. It has been suggested that LGI is the main cause of some diabetes-associated co-morbidities^4,6,7^. Recently, our group showed that LGI in diabetic mice depends on high levels of the lipid mediator leukotriene (LT) B4 in the blood and that LTB4 increases the adaptor molecule of the *toll-like* receptors family, MyD88, and its transcription factor STAT1 (Signal Transducer and Activator of Transcription 1). The chronic activation of *toll-like*/IL1β would then be responsible for the chronic systemic LGI^8^. Leukotrienes are generated from arachidonic acid (AA) through the action of the enzyme 5-lipoxigenase (5LO). The engagement of TLR/IL-1β receptors activates the enzyme phospholipase A2 which releases AA from membrane phospholipids. Through the action of the 5LO enzyme, AA is converted to LTA4, which can be rapidly converted to leukotriene LTB4 by specific enzymes^9,10^. In the present study, we will further investigate the role of leukotrienes in diabetes by inducing diabetes in mice lacking 5LO (5LO^−/−^).

One of the most important co-morbidities in diabetes is impaired healing. Macrophages are involved in all steps of the inflammatory process, from triggering to resolution^11,12^. These cells exhibit high plasticity and can differentiate into a spectrum of subpopulations, depending on microenvironment stimuli^13,14^. On one side of the spectrum, there are M1 macrophages, which possess a pro-inflammatory phenotype and microbicide activity, and on the other side are M2 macrophages, which possess an anti-inflammatory phenotype or repair activity. However, subtypes of the M2 phenotype were found; to simplify this, Fleming and Mosser (2011) proposed the following classification: a) classically activated (M1) macrophages which present a high expression of *Stat1*, *Nos*2 and *Il12*, and are often involved in immune response against infections; b) regulatory (M2) macrophages that comprise those characterized by high IL-10 production that down-regulate inflammation and immunity; and c) alternatively activated macrophages^15^, also M2, which present high expression of *Arg1*, *Fizz1* and *Ym1*, low expression of *Il10* and are involved in healing processes promoting angiogenesis, collagen deposition and wound closure^16–18^. Thus, adequate wound healing requires a continuum of phenotypic changes where, at the initial stage, classically activated macrophages are required for the clearance of invading microorganisms, dead neutrophils and tissue debris. Once the site is cleared, the need to dampen the inflammation leads to macrophages reprogramming towards alternative profile that will finally lead to tissue repair via cytokines and growth factors that stimulate cell proliferation, fibroblasts activation and extracellular matrix formation and neovascularization^19^. A prolonged inflammatory response would delay healing; in the diabetic condition, chronic LGI affects healing. In a mice model of diabetes, the wound healing process was accompanied by a long-lasting accumulation of classically activated macrophages which produce high levels of pro-inflammatory cytokines and low levels of growth factors^19,20^. Leukotrienes were shown to modulate macrophages phenotype in mice^8,10^, and the high levels of eicosanoids found in spontaneous ulcers in mice were reported to correlate with defective healing of the lesions^21^.

In the present study, we examined monocytes and macrophages phenotypes, and wound healing in diabetic mice that were sufficient or deficient in leukotrienes (5LO^−/−^). A better comprehension of the mechanisms involved in systemic inflammation and the impaired wound healing in diabetes could provide new strategies to improve the life quality of diabetics.

## Results

### 5LO products block M2 monocytes/macrophages polarization in diabetic mice

**Figure 1:**
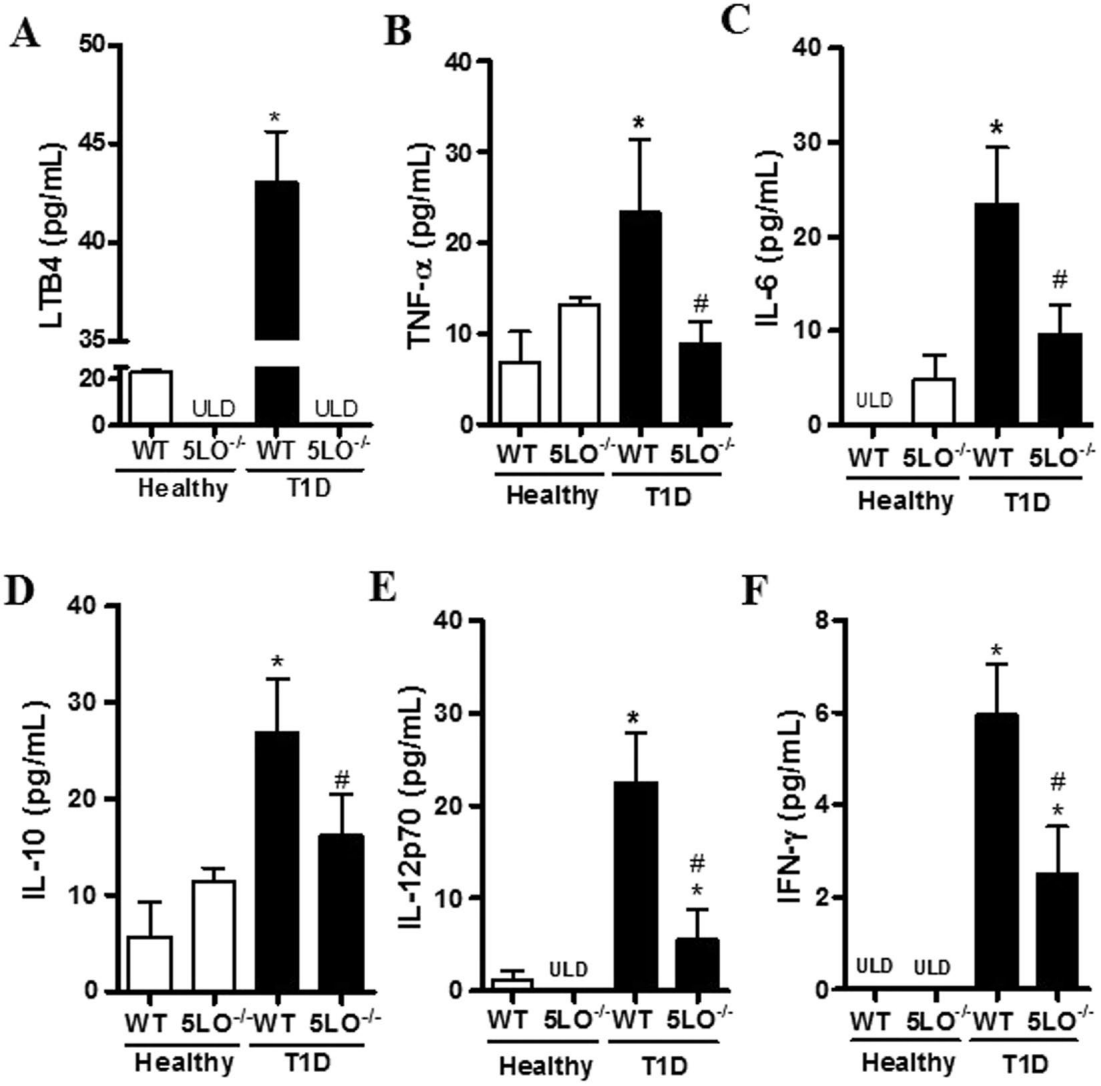
Systemic levels of LTB4 and cytokines in diabetic 5LO^−/−^ or WT mice. Plasma was collected from WT or 5LO^−/−^ mice, healthy or diabetic (T1D), for LTB4 dosage by ELISA (**A**). Serum from mice was collected for cytokine dosage by CBA: TNF-α (**B**), IL-6 (**C**), IL-10 (**D**) IL-12p70 (**E**) and IFN-γ (**F**). Data are presented as the mean ± SEM of 4 to 6 animals per group. *p ≤ 0.05 when diabetic groups were compared to healthy WT, and ^#^p ≤ 0.05 when diabetic 5LO^−/−^ mice were compared to diabetic WT. ULD = under the limit of detection.

We have previously shown that diabetic mice have increased systemic levels of pro-inflammatory IL-1β and TNF-α which are dependent on the increased LTB_4_ concentration found in the blood of the diabetic mice, since they were reduced by blocking LTB4 receptors^8^. Here, we compared the systemic concentration of LTB4 and cytokines in diabetic 5LO^−/−^ and WT mice and confirmed the higher concentration of LTB4 in the plasma of diabetic WT compared to healthy mice (Fig. 1A). Moreover, while the concentration of TNF-α, IL-6, IL-10, IL-12 and IFNγ in the serum of diabetic WT mice was increased compared to healthy WT, in the diabetic 5LO^−/−^ this increase was not observed (Fig. 1B–F).

**Figure 2:**
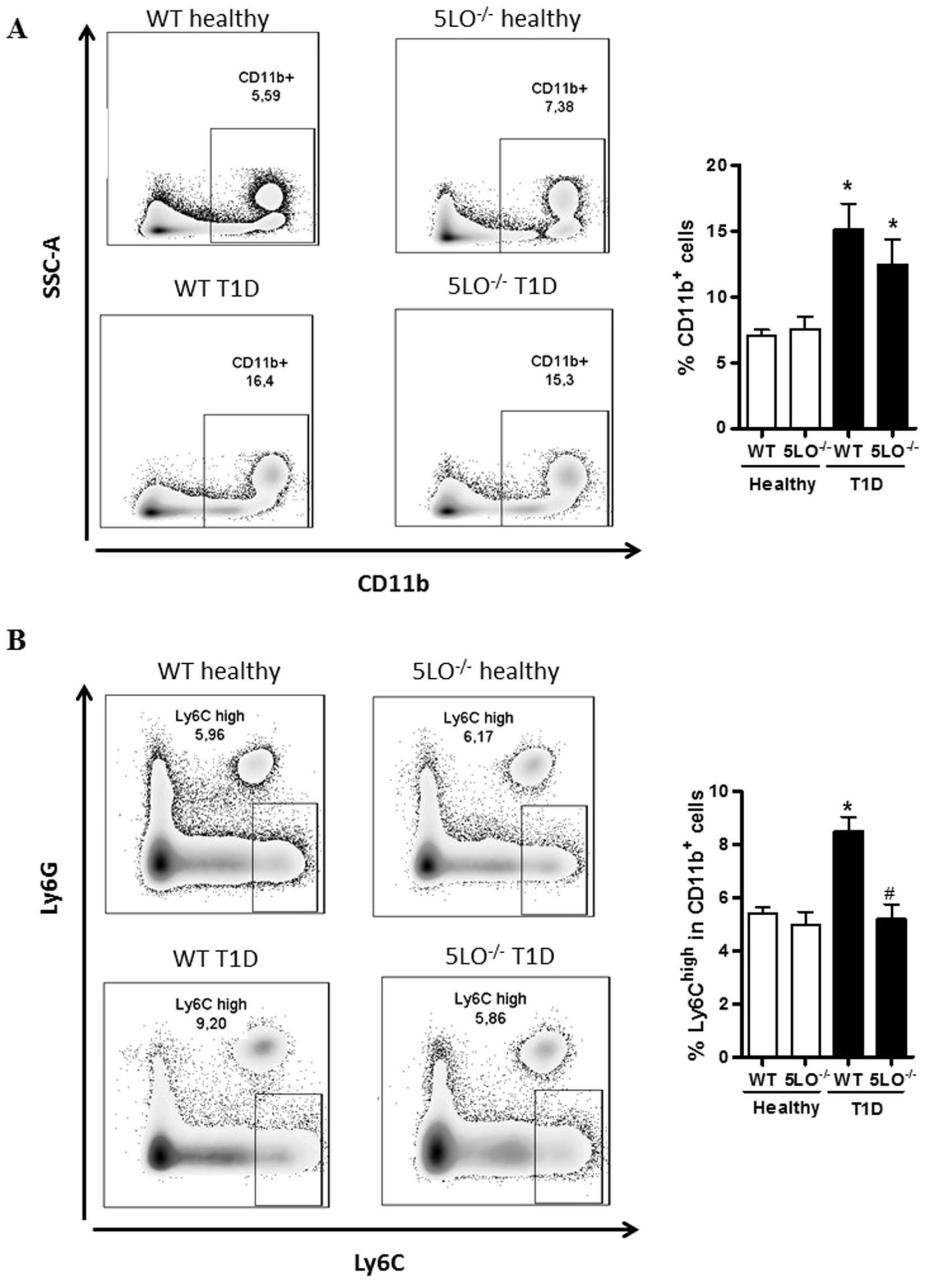
Phenotypic analysis of blood monocytes from diabetic 5LO^−/−^ or WT mice. Monocytes from WT or 5LO^−/−^ mice, healthy or diabetic (T1D) mice were isolated and prepared for monocyte isolation and preparation for the flux cytometer. The full gate strategy is shown in Supplementary Figure 1. CD11b^+^ cell frequency was determined (**A**). In this population, Ly6G^+^ were excluded, and only Ly6C^+^ were used for analysis (**B**). Data are presented as the mean ± SEM of 8 to 10 animals per group. *p ≤ 0.05 when diabetic groups were compared to healthy WT. ^#^p ≤ 0.05 when the diabetic 5LO^−/−^ group was compared to diabetic WT.

We next analyzed the phenotype of peripheral blood monocytes and found that whereas the diabetic WT mice presented an increased frequency of the pro-inflammatory monocytes (CD11b^+^Ly6C^high^Ly6G^−^) within the myeloid cells (CD11b^+^) population (Fig. 2A), in  the diabetic 5LO^−/−^ mice, this increase was not observed (Fig. 2B).

**Figure 3:**
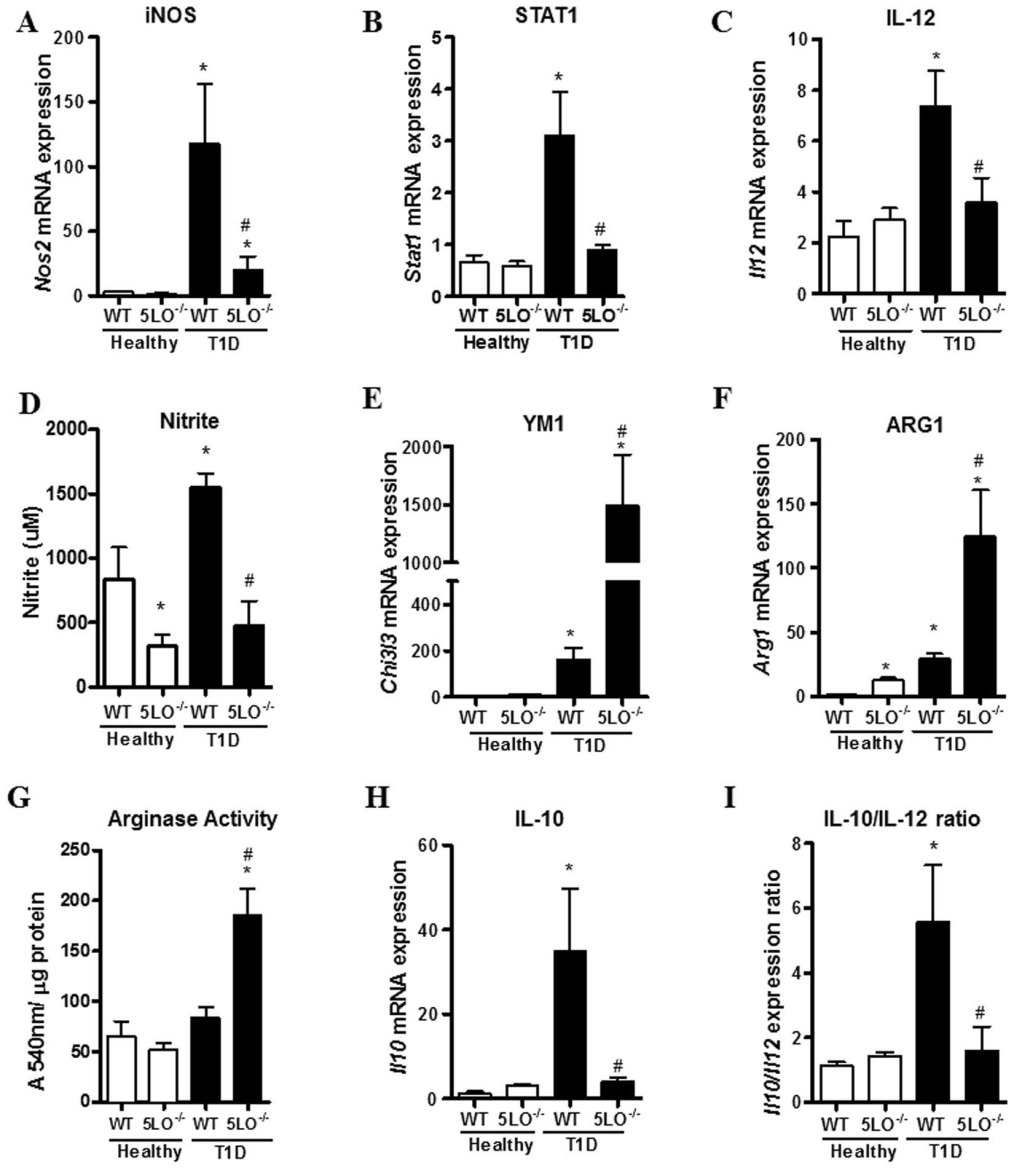
Phenotypic analysis of peritoneal macrophages from diabetic 5LO^−/−^ or WT mice. Resident peritoneal macrophages were collected from WT or 5LO^−/−^ mice, healthy or diabetic (T1D), for the analysis of macrophage phenotypic markers by qPCR. The markers analyzed were *Nos2* (**A**), *Stat1* (**B**), *IL12* (**C**), *Ym1* (**E**), *Arg1* (**F**), *Il10* (**H**), and *Il10/Il12* ratio (**I**). Functional assays for nitrite production (**D**) and arginase activity (**G**) were also performed in these cells. Data are presented as the mean ± SEM of 7 to 10 animals per group. *p ≤ 0.05 when diabetic WT group was compared to healthy WT, and ^#^p ≤ 0.05 when diabetic 5LO^−/−^ group was compared to diabetic WT.

Since pro-inflammatory monocytes can differentiate into M1 macrophages^22^, we next investigated the phenotypic markers expressed by peritoneal macrophages. We found that macrophages from diabetic mice compared to those from healthy mice presented increased gene expression of classically activated macrophages, such as iNOS (encoded by *Nos2* gene), *Stat1* and *Il12*, and the increased production of nitrite (Fig. 3A–D).

Conversely, macrophages from diabetic 5LO^−/−^ mice presented enhanced *Arg1* and *Ym1* (encoded by *Chi3l3* gene) expression and arginase production (Fig. 3E–G). Thus, in the absence of leukotrienes, macrophages from diabetic mice are compatible with the wound healing (alternative) phenotype.

**Figure 4:**
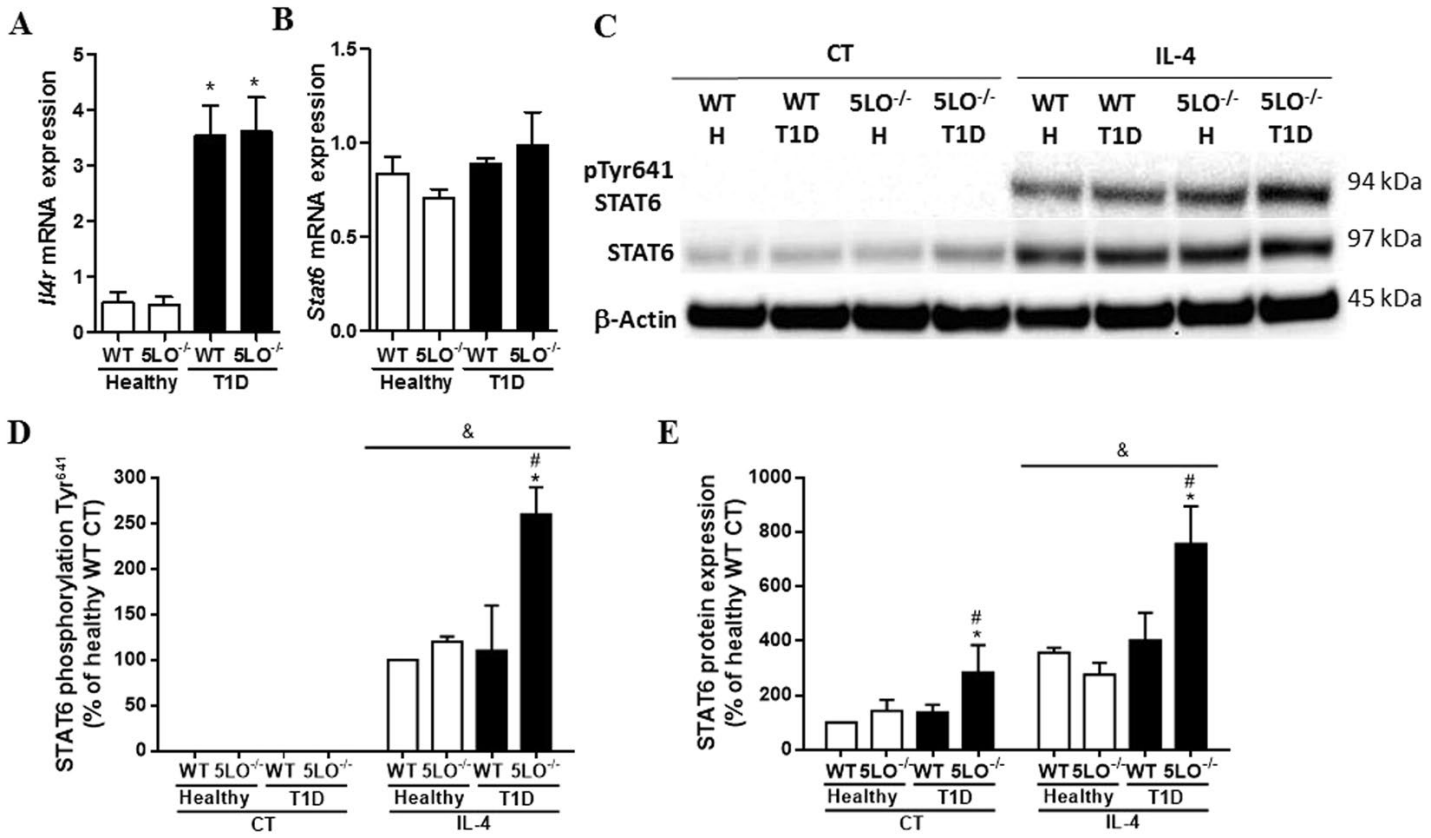
Evaluation of alternative pathway in macrophages from diabetic 5LO^−/−^ or WT mice. Resident peritoneal macrophages from WT or 5LO^−/−^ mice, healthy or diabetic (T1D), were collected for the analysis of *Il4r* (**A**) and *Stat6* (**B**) expression by qPCR. Total and phosphorylated STAT6 was also analyzed by western blot (**C**,**D**). Cells were stimulated with recombinant IL-4 (4000, 400 or 40 ng), and the expression of total STAT6 and phosphorylated (p-STAT6) and β-Actin were analyzed by western blot (**C**–**E**). Full-length blots/membranes are presented in Supplementary Figures 3 (loading control), 4 (total STAT6), 5 (p-STAT6) and 6 (β-Actin). Data are presented as the mean ± SEM of 3 to 5 animals per group. *p ≤ 0.05 when the diabetic groups were compared to healthy WT, ^#^p ≤ 0.05 when diabetic 5LO^−/−^ group was compared to diabetic WT. ^&^p ≤ 0.05 when IL-4 groups were compared to control (CT) groups. H = healthy.

**Figure 5:**
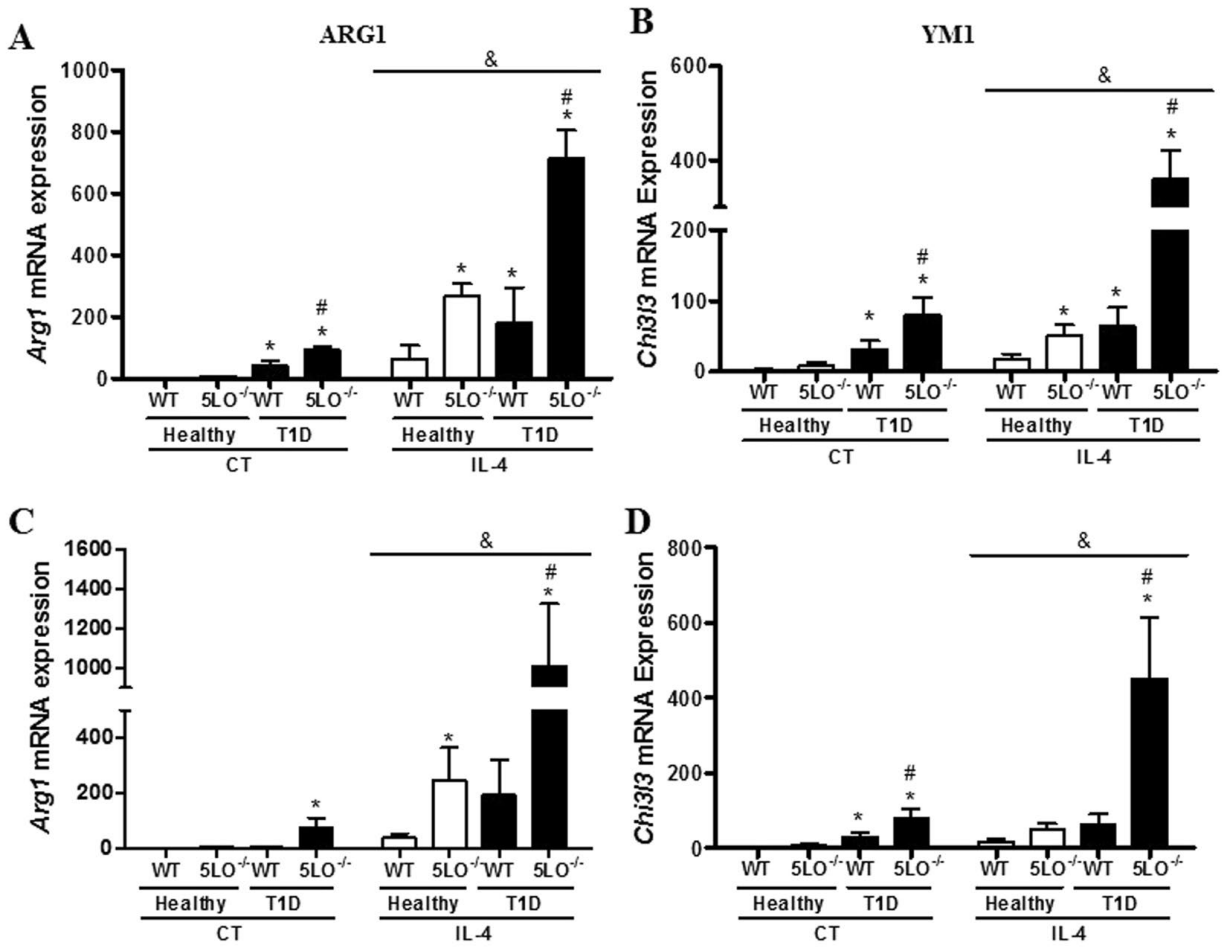
Effect of IL-4 on peritoneal macrophages from diabetic 5LO^−/−^ or WT mice. Macrophages obtained from WT or 5LO^−/−^ mice, healthy or diabetic (T1D), were stimulated with recombinant IL-4, and after 4 h gene expression of *Arg1* (**A**) and *Ym1* (**B**) were analyzed by qPCR. In another experiment, IL-4 was injected intraperitoneally, and after 4 h peritoneal macrophages were collected for gene expression analysis of *Arg1* (**C**) and *Ym1* (**D**). Data are presented as mean ± SEM of 4 to 5 animals per group. *p ≤ 0.05 when the groups were compared to healthy WT, ^#^p ≤ 0.05 when the diabetic 5LO^−/−^ group was compared to diabetic WT. ^&^p ≤ 0.05 when IL-4 groups were compared to control (CT) groups.

We next investigated whether the classically activated phenotype exhibited by diabetic mice could be reversed by stimulation with IL-4, a cytokine known to induce alternatively activated macrophages through the activation of STAT6. In non-stimulated macrophages, we first evaluated the expression of IL-4 receptors (encoded by *Il4r* gene) in resident peritoneal macrophages. IL4 receptor expression was found to be enhanced in diabetic mice, both WT and 5LO^−/−^ (Fig. 4A), and STAT6 mRNA expression was similar in all groups analyzed (Fig. 4B). When stimulated *in vitro* with IL-4, macrophages from diabetic 5LO^−/−^ mice showed enhanced STAT6 phosphorylation in Tyr^641^ compared to the other groups (Fig. 4C,D). Total STAT6 was more highly expressed in macrophages from diabetic 5LO^−/−^ mice, and IL-4 enhanced STAT6 protein expression in all groups, and still more in diabetic 5LO^−/−^ mice (Fig. 4C,E). As expected, an enhanced expression of the STAT6 product genes *Arg1* and *Chi3l3* was observed in the cells of all groups, and when the groups were analyzed separately, diabetic 5LO^−/−^ mice had a higher expression of *Arg1* and *Chi3l3* (Fig. 5A,B).

We then injected IL-4 intraperitoneally and measured *Chi3l3* and *Arg1* mRNA expression in resident peritoneal macrophages. IL-4 enhanced the expression of these genes in all groups, and when compared separately, macrophages from diabetic 5LO^−/−^ showed enhanced *Arg1* and *Chi3l3* mRNA expression compared to diabetic WT mice (Fig. 5C,D).

Taken together, these data suggest that under diabetic conditions, 5LO products play important roles in monocytes and macrophages phenotype.

### 5LO products are involved in the impaired wound healing in diabetic mice

**Figure 6:**
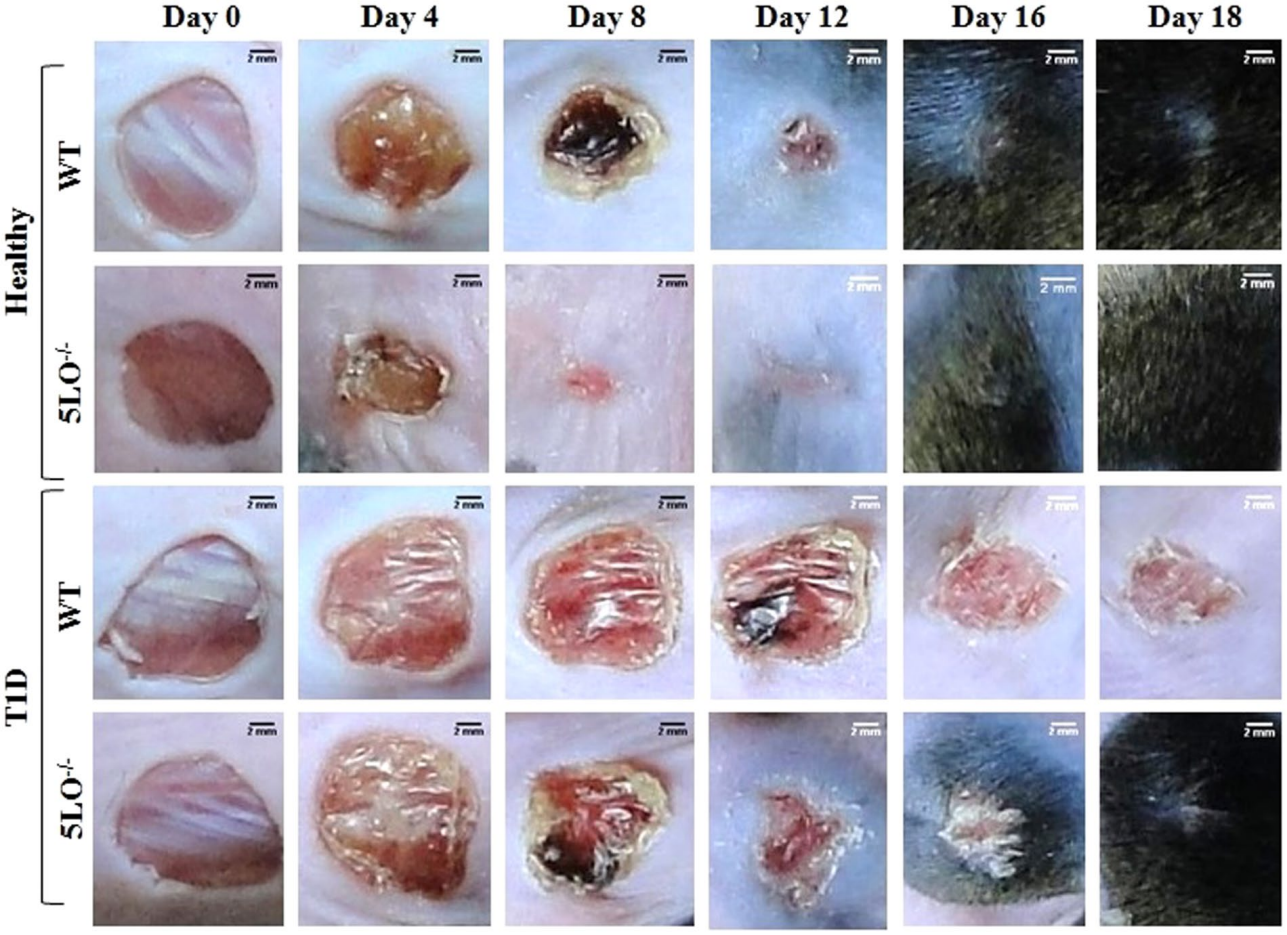
Temporal evaluation of wound healing in diabetic 5LO^−/−^ or WT mice. WT or 5LO^−/−^ mice, healthy or diabetic (T1D), were submitted to a full thickness skin lesion model, and lesions were photographed on alternate days at a 20 cm distance from animal back. The assay was performed using 4 to 8 mice per group, in 2 independent experiments.

**Figure 7:**
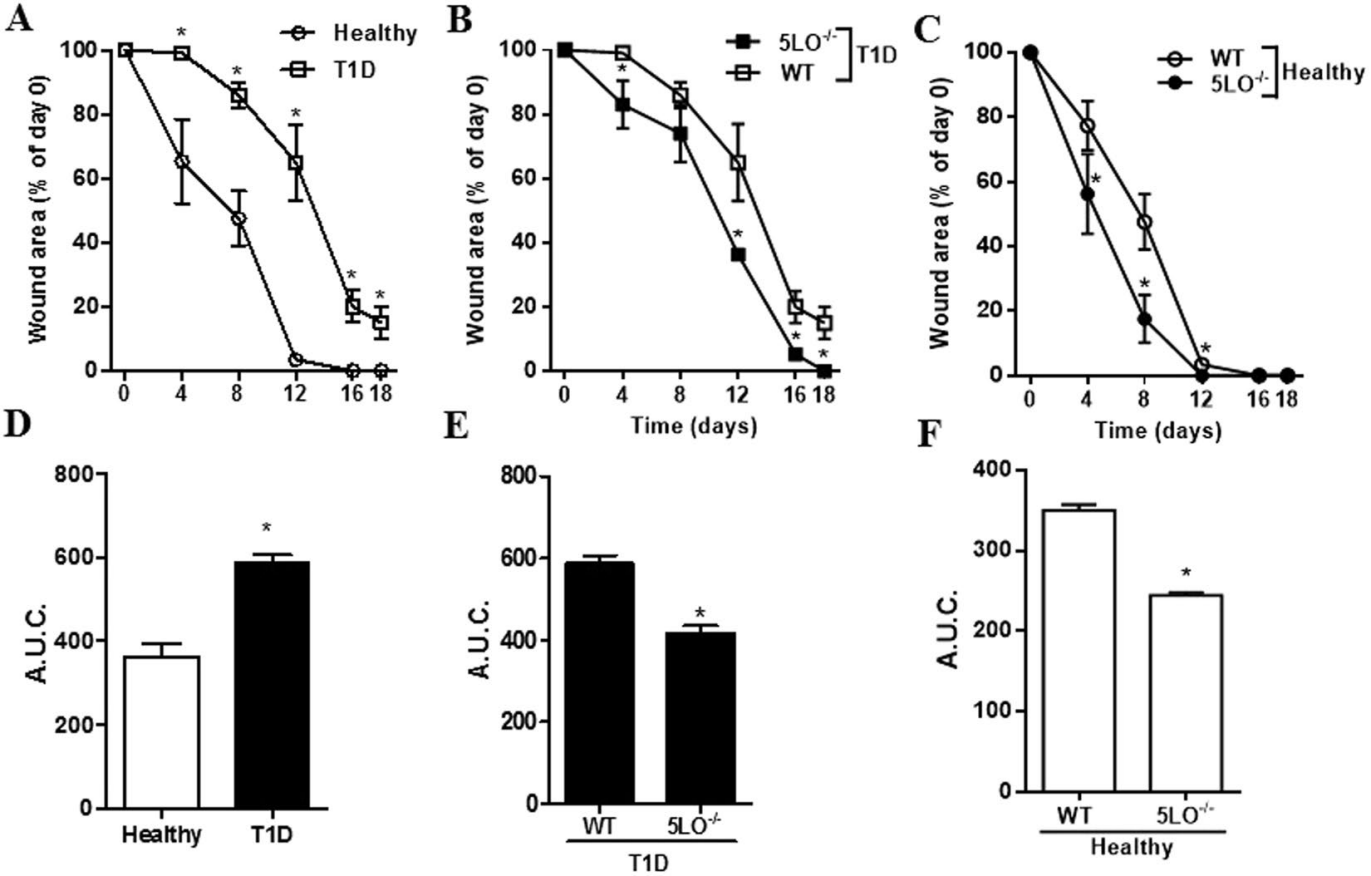
Lesion area measurement in wounds from diabetic 5LO^−/−^ or WT mice. Lesion areas from WT or 5LO^−/−^ mice, healthy or diabetic (T1D), were calculated using Image J and the measurements obtained in mm on Day 0 were considered 100% of the lesion (**A**–**C**). Area under curve (AUC) analyses were performed according to data from timeline graphs (**D**–**F**). Data are presented as the mean ± SEM of 4 to 8 animals per group in 2 independent experiments. *p ≤ 0.05 when the diabetic WT group was compared to healthy WT.

In some diabetic patients, wound healing is impaired^23,24^. Since alternatively activated M2 macrophages are involved in wound healing^12^, and we found that macrophages from diabetic 5LO^−/−^ mice exhibit this phenotype, we investigated the wound healing in diabetic mice after the full thickness excision of lesions. In morphological macroanalysis (Fig. 6), similarly to humans, diabetic mice presented delayed wound closure. At all times examined, healthy wounds were significantly smaller than those in diabetics (Fig. 7A). Area under curve also represented this difference (Fig. 7D). On Day 18, the diabetic lesions were closed in 5LO^−/−^ mice with hair growth in the surrounding area, while the lesions in the diabetic WT mice were still around 15% of the original size and no hair growth in the surrounding area was observed (Figs 6, and 7B). The impairment of wound closure in diabetics was less pronounced in the 5LO^−/−^ mice, different from that in diabetic WT. This was observed in the measurements of wounds area and area under curve (Fig. 7E). In healthy mice, the 5LO deficiency significantly and positively affected wound healing. By morphological analysis, the wound closure was faster in 5LO^−/−^ mice (Fig. 6) and wounds area calculations show that this difference is significant on days 4, 8, and 12, as confirmed in the area under curve graph. On day 8, healthy 5LO^−/−^ mice presented only 17% of the original lesion size, while healthy WT mice lesions had a size of almost 50%. On day 12, however, lesions of healthy 5LO^−/−^ mice were already closed, while lesions of healthy WT mice were around 4% of the original size (Fig. 7C,F).

**Figure 8:**
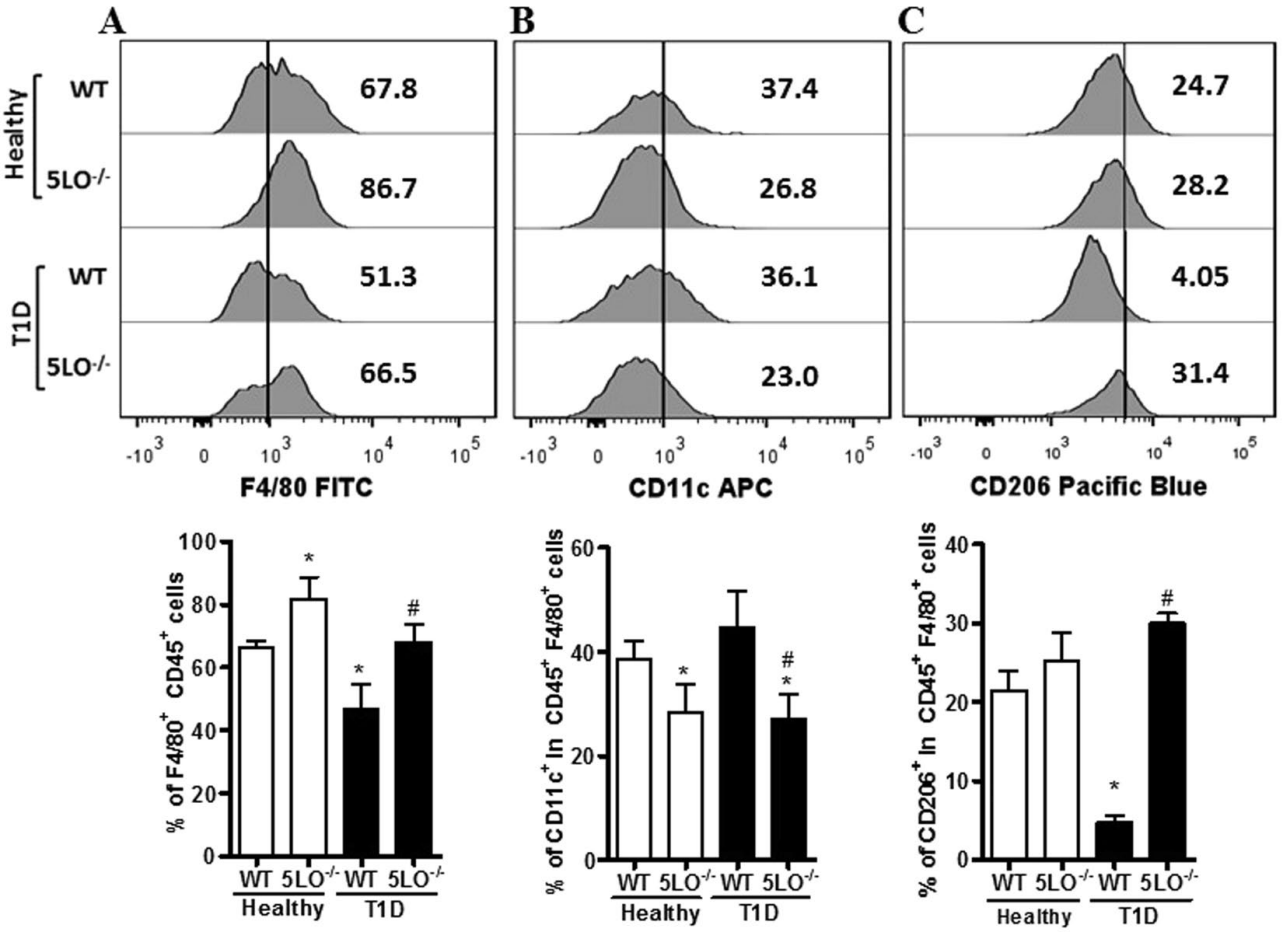
Phenotypic analysis of resident wound macrophages from diabetic 5LO^−/−^ or WT mice. On day 3 after wound induction, the lesions from WT or 5LO^−/−^ mice, healthy or diabetic (T1D), were collected and processed for cell isolation. Full gate strategy is in Supplementary Figure 2. Macrophages frequency was determined by % of F4/80^+^CD45^+^ cells (**A**,**D**), and the frequency of macrophages with different phenotypes was determined by % of CD11c^+^ (**B**,**E**) cells or % of CD206^+^ (**C**,**F**), respectively, in the F4/80^+^CD45^+^ population. Data are presented as the mean ± SEM of 6 animals per group. *p ≤ 0.05 when diabetic groups were compared to healthy WT. ^#^p ≤ 0.05 when diabetic 5LO^−/−^ were compared to diabetic WT.

We next investigated whether the defective wound healing in diabetic WT mice is due to changes in the wound macrophages phenotype. On day 3 after wound induction, the skin lesions were collected and processed for cell isolation. Healthy and diabetic 5LO^−/−^ mice presented higher frequency of macrophages (CD45^+^F4/80^+^) in the wound when compared to the other groups (Fig. 8A); however, the frequency of M1 macrophages (CD45^+^F4/80^+^CD11c^+^) was lower in both healthy and diabetic 5LO^−/−^ mice (Fig. 8B), and the frequency of M2 macrophages (CD45^+^F4/80^+^CD206^+^) was reduced only in the wounds of diabetic WT mice (Fig. 8C). In this assay, we also analyzed the mean fluorescence intensity (MFI), and the results followed the same pattern observed in the percentage of positive cells frequency (Supplementary Table 1). This suggests that the defective healing in diabetic WT mice is due to the decreased frequency of alternatively activated macrophages in the wound.

Together, these results suggest that leukotrienes hinder the polarization of the classic to the alternative macrophages phenotype in diabetic mice, which correlates with the impaired wound healing in diabetics.

## Discussion

Diabetes is a metabolic disorder characterized by chronic LGI. Studies have reported that this inflammatory status depends on the increased systemic levels of LTB4 found in diabetic mice^8,25^.

We aimed to investigate the relationship between LGI setting, increased levels of LTB4, and how this would affect the wound healing process in diabetic mice. We found that in peripheral blood from diabetic mice, the increased levels of cytokines (IFN-γ, IL-12, IL-6, TNF-α and IL-10) were dependent on leukotrienes as well as monocytes reprogramming towards a pro-inflammatory phenotype characterized by the increased frequency of the CD11b^+^Ly6C^high^ population. Resident peritoneal macrophages from diabetic WT mice bear a classically activated-like phenotype (higher *Stat1* and *Nos2* expression, and higher NO levels), while macrophages from diabetic 5LO^−/−^ mice exhibit an alternatively activated phenotype (higher *Arg1* and *Ym1* expression, and higher activity of arginase). In the absence of leukotrienes, the enhanced phosphorylation of STAT6, and increased gene expression of STAT6 product genes (*Arg1*, *Chi3l3*) were observed when macrophages were stimulated with IL-4. Skin wound healing in diabetic WT mice was impaired and this correlated with the decreased frequency of alternatively activated macrophages (CD45^+^F4/80^+^CD206^+^) in the lesions.

Systemic inflammation in diabetes has been associated with complications which are common in diabetic patients, such as retinopathy^7^ and endothelial dysfunction^25^. In STZ-induced diabetes, increased cytokines levels in the blood are observed in WT but not in 5LO^−/−^ mice. This suggests that leukotrienes have a central role in the systemic setting in diabetes. This is in accordance with previous results in which high systemic levels of IL-1β in diabetic mice were not observed in mice lacking the LTB4 receptor BLT-1 or in animals treated with a 5LO inhibitor^8^. Due to LTB4 amplification of MyD88-dependent responses, we suggest that cytokine production in diabetes occurs in response to the activation of receptors that use MyD88 as an adaptor molecule to activate the NFκB pathway, the TLR family. It is thus possible to speculate that leukotrienes would also contribute to the systemic inflammation.

In addition to the high cytokine levels in diabetes, there is also a higher frequency of pro-inflammatory monocytes (CD11b^+^Ly6C^high^), and both events are leukotrienes-dependent. This finding is in accordance with Spite *et al*.^26^, who showed that high fat diet-induced diabetic mice have a higher frequency of these cells in an LTB4–dependent manner; this was not seen in mice lacking BLT1. Moreover, Yang *et al*.^27^ suggested that the high frequency of CD11b^+^ Ly6C^high^ in PBMC could be used as a biomarker in inflammatory diseases.

CD11b^+^Ly6C^high^ monocytes can migrate from blood to the peritoneal cavity in systemic inflammation^28^, where they become classically activated macrophages (higher *Stat1* and *Nos2* expression, and higher production of NO), as observed in diabetic mice. Surprisingly, macrophages from diabetic mice also expressed Il10. According to the classification of Fleming and Mosser (2011) the M2 phenotype comprises the alternatively activated macrophages, which present high expression of *Arg1*, *Fizz1* and *Ym1*, and are involved in healing process. The regulatory macrophages are characterized by high *Il10* and low *Il12* that down regulate inflammation. In the experimental model of type 1 diabetes employed in this study, the insulin producing cells in pancreas are destroyed by streptozotocin that is injected into the peritoneal cavity^29^. This should induce an acute inflammation and, by the time macrophages were collected for PCR analysis (10 days after last streptozotocin injection), besides the classically activated macrophages, it is expected that there is already a population of regulatory macrophages expressing Il10 to down regulate the initial inflammation induced by streptozotocin. Interestingly, this was not observed in 5LO^−/−^ diabetic mice suggesting that the IL10 production is partially dependent on 5LO products in WT diabetics. Indeed, blockade of lipoxin A4 (a product from 5LO metabolism) synthesis, prevents IL-10 production and this is accompanied by partial reversion of inflammation^30^.

In diabetic 5LO^−/−^ mice, the macrophage profile was similar to the alternatively activated phenotype (high *Ym1* and *Arg1* expression, higher arginase activity)^15^. The *Arg1* marker encodes the enzyme arginase, which converts arginine into ornithine. Ornithine is involved in the extracellular matrix development^18^, while chitinases, encoded by the *Ym1* gene, have a carbohydrate and matrix binding role^16^. All of these molecules are known to be involved in wound healing. Due to the low expression of *Il10*, and high levels of healing markers, we suggest that diabetic 5LO^−/−^ macrophages have a phenotype similar to the alternatively activated macrophages.

According to Mosser and Zhang^31^, classically activated macrophages inhibit alternative activation in some experimental models. We stimulated macrophages with recombinant IL-4, a cytokine which induces alternative activation, and evaluated the activation of the main transcription factor in this status, STAT6^32^. In the absence of leukotrienes, pSTAT6 was more strongly expressed; the products of STAT6 activation, *Ym1* and *Arg*, were more strongly expressed when the macrophages were stimulated with IL-4. Although macrophages from diabetic WT mice increased the expression of STAT6 and its products when stimulated with IL-4, this increase was not as high as in macrophages from diabetic 5LO^−/−^ mice. These data suggest that under diabetic conditions 5LO products hinder alternative macrophage polarization.

It is known that wound healing complications are recurrent in diabetic patients. A study showed that wound healing was defective in mice with insulin resistance induced by a high fat diet. In these mice, wound closure was delayed when compared to healthy animals, and this occurred in parallel with the disordered migration of fibroblasts to the wound site, and disorganized collagen deposition^33^. In our model, diabetic mice are insulin resistant (not published). Thus, corroborating with previous studies, we observed that diabetic mice have delayed wound healing when compared to healthy animals^33^. We also observed that diabetic 5LO^−/−^ mice had a faster wound closure than diabetic WT mice, and also faster hair growth in the surrounding area.

When we analyzed the healthy groups, 5LO^−/−^ mice healed sooner than healthy WT on days 4, 8 and 12 after excision and, on day 12, 5LO^−/−^ wounds were already closed, while WT wounds were still opened. Calculation of the area under curve showed that the difference is significant (p < 0.01). This is in accordance with a recent study, in which healthy 5LO^−/−^ mice presented faster wound closure and reduced inflammatory infiltrate in the skin, together with increased CD4 regulatory T cells markers in the draining lymph nodes. This study also showed that 5LO^−/−^ wounds also had diminished expression of pro-inflammatory cytokines and chemokines in the lesions, besides differential extracellular matrix remodeling^32^. Another study showed that 5LO^−/−^ mice accelerated cutaneous wound healing because in the absence of 5LO, heme oxygenase-1, which has anti-inflammatory properties, is upregulated^34^. This suggests that inhibition of 5LO can be used as a strategy in treating chronic lesions not only in diabetic condition, but also in other wounds types, as in large burns.

From the inflammatory phase until resolution, macrophages play a critical role, undergoing dynamic changes during the different phases. In the early stages of healing, classically activated macrophages mediate tissue damage and exert pro-inflammatory activities. In the later stages, the switch toward alternatively activated macrophages is involved in starting tissue repair and regeneration^12^. It has been suggested that healing is delayed in diabetics due to a prolonged accumulation of classically activated macrophages in the wounds, associated with elevated levels of pro-inflammatory cytokines^35^.

In healthy wounds, among various markers, the mannose receptor (CD206) is highly expressed^32,36,37^, and the CD11c marker has been reported in chronic wounds^38^. In our study, the wound microenvironment of WT mice had high levels of macrophages polarized to a classic profile (CD45^+^F4/80^+^CD11c^+^), and in mice lacking leukotrienes, the frequency of these cells is lower. The frequency of cells with the alternative activation marker (CD45^+^ F4/80^+^CD206^+^), however, is only lowered in diabetic WT mice (Fig. 8). This result suggests that the impaired wound healing in WT diabetic condition correlates with decreased frequency of alternatively activated macrophages in the wound.

Putting all of the data together, we propose that leukotrienes in diabetic mice amplify the sterile inflammation by increasing pro-inflammatory cytokine production. The inflammatory status, in turn, promotes monocytes activation, and macrophage reprogramming towards a classical-like activated profile. Since wound healing depends on a timely switch from a classic to alternative program in macrophages, we propose that the delayed wound healing in diabetic mice is due to deficient alternative macrophages in their wounds.

## Material and Methods

### Animals

Eight-to-ten week-old male 129 SvE and 5LO^−/−^ mice were maintained according to the National Institute of Health guidelines for the use of experimental animals with the approval of the Care of Animals and Ethical Committee for Animal Research of the Institute of Biomedical Sciences of the University of Sao Paulo. The animals were kept in micro-isolator cages under specific pathogen-free conditions.

### Induction of Diabetes Mellitus

Type 1 diabetes was chemically induced via five sequential daily intraperitoneal injections of a freshly prepared solution of streptozotocin (STZ 60 mg/kg) after 5 hours of food deprivation^8^. Blood glucose concentrations were measured 10 days after the last injection of STZ using One Touch Select glucometer and test strips (Life Scan). Mice were considered diabetic when blood glucose concentrations reached >300 mg/dL. The control group received five intraperitoneal (i.p.) injections of the vehicle, citrate buffer pH 4.5.

### Enzymatic assays

Serum was collected from mice for the dosage of cytokines TNF-α, IL-6, IL-10, IL-12 and IFN-γ using an Inflammation CBA kit (BD Biosciences, San Diego, CA, USA), following the manufacturer’s protocols. Plasma was collected from mice for the dosage of LTB4 using an LTB4 ELISA Kit (Cayman).

### Analysis of monocytes activation

Whole blood samples were collected from mice by cardiac puncture with a syringe containing 1% heparin (Sigma), and erythrocytes were lysed using AcK (5 M – Gibco by Life Technologies). After lysis protocol, cells were then resuspended in a FACS buffer (PBS containing 0.1% sodium azide and 1% FBS) and stained with fluorescent-conjugated monoclonal antibodies anti-Ly6G-PECy7, anti-Ly6C-PE (both eBioscience), and anti-CD11b-FITC (BD Pharmingen). Results were collected for 10,000 events and analyzed using FlowJo software (TreeStar). In the full gate strategy, doublets, debris and dead cells were gated out. First, CD11b^+^ were gated and Ly6C^high^and Ly6G^+^populations were determined in CD11b^+^ cells.Ly6G^+^ populations were excluded from the gate in the final analysis (see Supplementary Fig. S1).

### Peritoneal macrophages profile

Peritoneal exudates cells were obtained by lavage of the peritoneal cavity with cold PBS. The lavage fluid was centrifuged at 400 *g* for 10 min at 4 °C. The cells were cultured with RPMI 1640 medium, 100 μg/mL streptomycin, 60 units/mL penicillin, 11 mM sodium bicarbonate, 2 mM L-glutamine and 20 mM HEPES. Cells were left to adhere on a 6 well plate for 4 h at 37 °C in 5% CO_2_. Non-adherent cells were removed via aspiration of the supernatant and replacement with fresh RPMI 1640/1% FBS for 1 hour. After this incubation, cells were washed with PBS at 37 °C, and TRIzol was added for RNA extraction and the analysis of RNA expression of macrophages profile markers.

### Nitrite and arginine measurements

Macrophages were isolated by adherence for arginase activity measurements. The supernatant was collected for NO dosage and macrophages were scraped and lysed for arginase activity, as described previously^39^. Urea concentration was determined spectrophotometrically by the absorbance at 550 nm in a microplate reader (Molecular Devices, Sunnyvale, CA). The amount of urea produced was used as an index for arginase activity.

To evaluate NO production, nitrite concentration in the supernatants of macrophage cultures was measured using the standard Griess reaction^40^. Absorbance was measured at 540 nm using a 620-nm reference filter in a microplate reader and the nitrite concentration was calculated using a standard curve of sodium nitrite.

### Analysis of macrophages stimulated with IL-4

An assay of dose versus response was performed *in vivo* in resident peritoneal macrophages from healthy WT mice. In this assay, IL-4 (4000, 400, 40, or 4 ng) was intraperitoneally injected in 250 µL of PBS. *Arg1* was more highly expressed in macrophages from mice that received 40 ng of IL-4 (Supplementary Fig. S2). Resident peritoneal cells were collected by peritoneal lavage 4 h after IL-4 or PBS intraperitoneal injection. A pool of 2 mice/group was made. The lavage was collected and centrifuged, and pellets containing cells were plated on 6-well plates. After 4 h, non-adhered cells were washed with PBS, and TRIzol was added for posterior gene expression analysis.

In another experiment, peritoneal lavage was collected from mice for IL-4 stimulation *in vitro*, and the previous procedures were undertaken. After washing non-adherent cells, the macrophages were stimulated with IL-4 (40 ng) or media for 30 min. After stimulation, the supernatant was discarded and RIPA buffer containing proteases and phosphatases was added for protein extraction and the analysis of protein activation by western blot. In another experiment, the same protocol was made, but TRIzol was added to the cells for posterior gene expression analysis 4 h after stimulation with IL-4.

### Skin wound induction and closure assessment

A full-thickness excision lesion model was realized in mice anesthetized intraperitoneally with ketamin and xylazine (100 mg/kg and 10 mg/kg, respectively). Dorsal superficial fur was removed with shaving cream and asepsis was appled with 70% alcohol. The lesion was induced with an 8 mm^2^ biopsy punch. Mice wounds were photographed under isoflurane anesthesia on alternative days until the 20^th^ day after induction. Wound areas were measured using Image J Software (National Institutes of Health, Bethesda, MD), and areas obtained on day 0 were considered 100% of the lesion. The graphs present the mean according to all analyzed animals per group.

### Cell isolation from wound and flow cytometry

The dorsal dermis was scraped off on the 3rd day after wounding, and was enzymatically digested using collagenase I (450 U/mL), collagenase XI (125 U/mL), and hyaluronidase I-s (60 U/mL) in 20 mM HEPES. Cell suspensions were sieved with cold PBS and the cells were labeled with fluorescent-conjugated monoclonal anti-CD45-PE (BD Biosciences), -F4/80-FITC, -CD11c-APC, and -CD206-PB (Biolegend) antibodies. The labeled cells were analyzed using flow cytometry on a FACS Canto (BD Bioscience). Autofluorescence was determined using unlabeled cells from skin, and compensation was performed using cells from skin individually marked with the antibodies (BD Bioscience). The histograms of positive single-color stained cells were analyzed against a histogram of unstained cells to show where positive gates for CD45, F4/80, CD11c and CD206 were located (Supplementary Fig. S3). About 10,000 events were analyzed^41^. In the full gate strategy, doublets, debris and dead cells were gated out. First, CD45^+^ cells were gated, and F4/80^+^ were gated inside the CD45^+^ population. Then, CD11c^+^ or CD206^+^ cells were selected inside the CD45^+^F4/80^+^ population (see Supplementary Fig. S4). Mean fluorescence intensity (MFI) was also observed in the final gates (Supplementary Table 1). All antibodies used in this study were monoclonal. This dismisses isotype controls. All analysis considered automatic compensation using beads (BD Biosciences) in the cytometer before sample acquisition.

### RNA purification and analysis by qPCR

The total RNA from macrophages was isolated using Direct-zol RNA MiniPrep (Zymo Research) according to the manufacturer’s instructions. Complementary DNA (cDNA) was synthesized using a reverse transcription system (RevertAid First Strand cDNA Synthesis Kit, Thermo Scientific) and qPCR was performed with Fast SYBR® Green Master Mix (Applied Biosystems) containing primers for *Ym1*, *Arg1*, *Nos2*, *Stat1*, *Stat6*, *Il10*, *Il12*, *Hprt* (all from Integrated DNA Technologies) (Table 1) on the Step One Plus Real-Time PCR Detection System (Applied Biosystems). Relative expression was calculated using the comparative threshold cycle (Ct) and calculated relative healthy WT (ΔΔCt method). The sequences of the primers are listed in Table 1.

**Table 1:** Sequence of primers.

Gene	Primer sequence
*Chi313*	5′-ACTGGTATAGTAGCACATCAGC-3′
	5′-AGAAGCAATCCTGAAGACACC-3′
*Arg1*	5′-AGTGTTGATGTCAGTGTGAGC-3′
	5′-GAATGGAAGAGTCAGTGTGGT-3′
*Nos2*	5′-CACTTCTGCTCCAAATCCAAC-3′
	5′-GACTGAGCTGTTAGAGACACTT-3′
*Stat1*	5′-GACTTCAGACACAGAAATCAACTC-3′
	5′-TTGACAAAGACCACGCCTT-3′
*Stat6*	5′-AGTTCTTCCTGCTTCCGATG-3′
	5′-GCCACCATCAGACAAATACTTC-3′
*Il10*	5′-ATGGCCTTGTAGACACCTTG-3′
	5′-GTCATCGATTTCTCCCCTGTG-3′
*Il12*	5′-TGGTTTGCCATCGTTTTGCTG-3′
	5′-ACAGGTGEGGTTCACTGTTTCT-3′
*Il4r*	5′-CCTCACACTCCACACCAATG-3′
	5′-TCTGCAGGGTTGTCCTCTCT-3′
*Hprt*	5′-AGCAGGTCAGCAAAGAACT-3′
	5′-CCTCATGGACTGATTATGGACA-3′

### Western Blot

Protein samples derived from macrophages stimulated with IL-4 *in vitro* or *in vivo* were obtained with RIPA buffer. The samples were submitted for electrophoresis in SDS-PAGE, and were transferred to the nitrocellulose membrane. After transfer, the membranes were incubated with primary antibodies anti-STAT6, -pSTAT6 (Abcam), and -β-actin (Cell Signaling). The membranes were then incubated with proper secondary antibody HRP-conjugated. At the end of incubation, SuperSignal West Pico Cheluminescent Substrate was used to detect HRP according to the manufacturer’s instructions. Full-length blots/membranes are presented in Supplementary Figures 3 (loading control), 4 (total STAT6), 5 (p-STAT6) and 6 (β-Actin).

### Statistical analyses

The results are presented as mean ± standard error of the mean from at least three independent experiments, and were analyzed with GraphPad Prism Software 5.0 (San Diego, CA, U.S.A.) by analysis of variance (ANOVA) followed by a Bonferroni analysis. Differences were considered significant when p ≤ 0.05.

## Electronic supplementary material

Supplementary Information

## Data Availability

All data generated or analysed during this study are included in this published article (and its Supplementary Information files). All data are available from the corresponding author on reasonable request.
