# Supplementary material for: Impaired wound healing in type 1 diabetes is dependent on 5-lipoxygenase products
Source: Sci Rep. 2018 Sep 21;8:14164. doi: 10.1038/s41598-018-32589-7 (PMC6155046; doi:10.1038/s41598-018-32589-7)
Supplement: Supplementary file 1 — Supplementary Information [file 41598_2018_32589_MOESM1_ESM.pdf]

Impaired wound healing in type 1 diabetes is dependent on 5-lipoxygenase products

Theresa Ramalho[1], Luciano Filgueiras[1], Ildefonso Alves Silva-Jr[1], Ana Flavia Marçal Pessoa[2], Sonia Jancar[1]*

[1]Department of Immunology, Institute of Biomedical Sciences, University of São Paulo, São Paulo, Brazil

[2]Department of Cell and Developmental Biology, Institute of Biomedical Sciences, University of São Paulo, Sao Paulo, Brazil

*Correspondence address: Department of Immunology, Institute of Biomedical Sciences, University of São Paulo. Av. Prof. Lineu Prestes, 1730, 05508-900, Butantã, São Paulo, SP, Brazil. E-mail: sojancar@icb.usp.br

SUPPLEMENTARY INFORMATION

Supplementary table 1. Values of mean fluorescence intensity in the final gates used in flow cytometer analysis of cells isolated from wounds.

Groups	F4/80	CD11c	CD206
**Healthy WT**	1337	808	3547
**Healthy 5LO$^{-/-}$**	1606	660	3808
**Diabetic WT**	1018	777	2578
**Diabetic 5LO$^{-/-}$**	1317	599	4125

Supplementary Figure 1

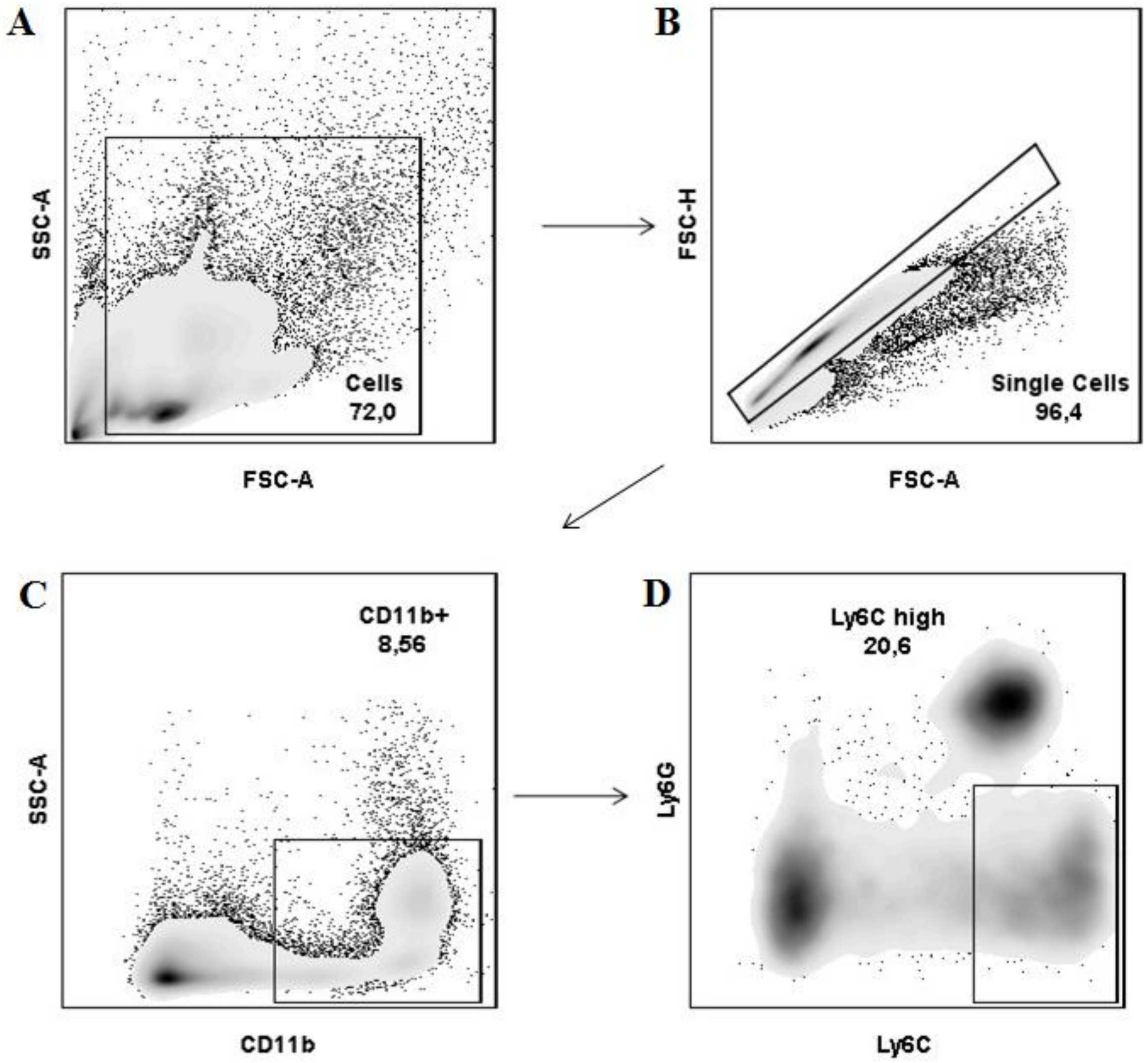

Supplementary Figure 1. Full gate strategy for analysis of peripheral monocytes. Peripheral blood was collected from WT or 5LO$^{-/-}$ mice, healthy or diabetic (T1D) mice for monocyte isolation and preparation for the flux cytometer. Debris, dead cells (A), and doublets (B) were gated out; the CD11b^{+} population was determined (C). Ly6C^{high} and Ly6G^{+} and populations were determined in CD11b^{+} cells. Ly6G^{+} populations were excluded from the final analysis.

Supplementary Figure 2

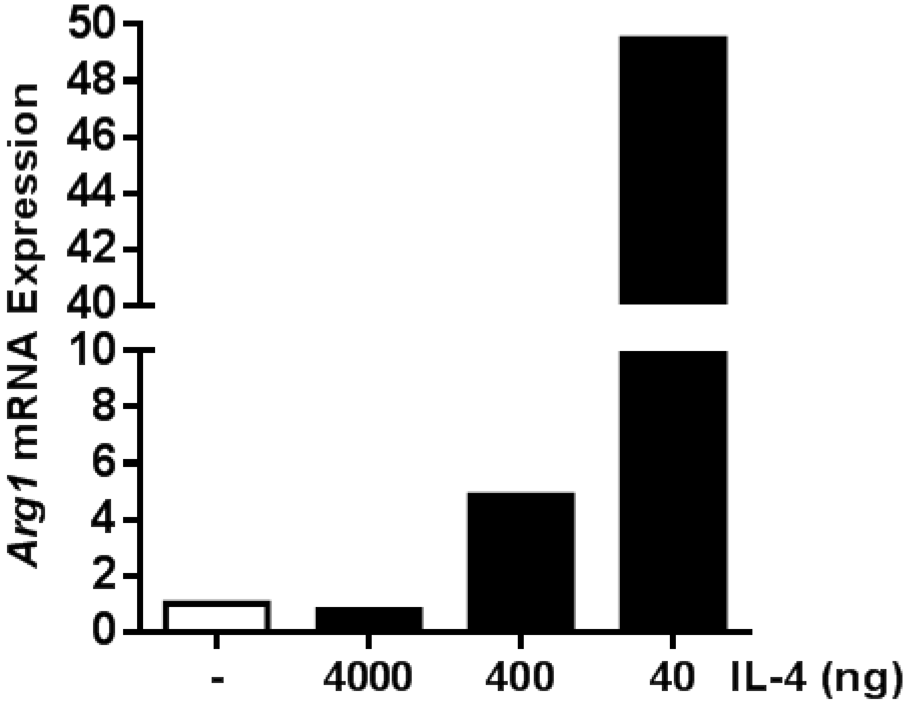

Supplementary Figure 2. Dose response in macrophages stimulated with IL-4. Healthy WT mice were injected intraperitoneally with IL-4 in a range of different doses. After 4 hours, resident peritoneal macrophages were collected and processed for analysis of *Arg1* gene expression by qPCR.

Supplementary Figure 3

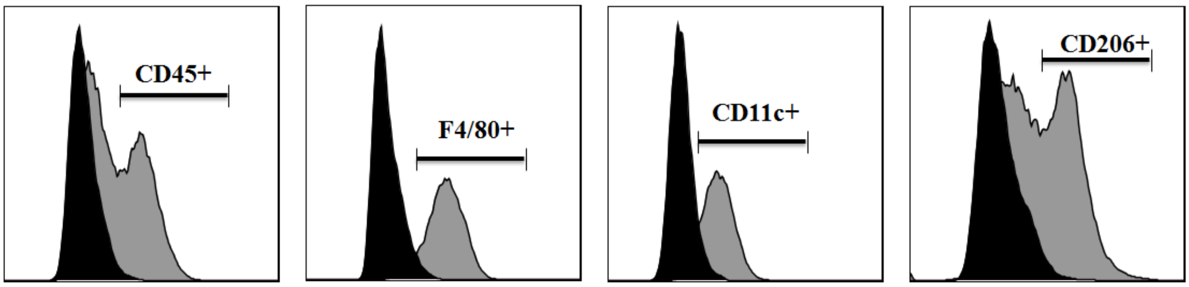

Supplementary Figure 3. Positive gates for stained cells excluding cell autofluorescence. The lesions from WT or 5LO$^{-/-}$ mice, healthy or diabetic (T1D), were collected and processed for flow cytometry. Positive single-color stained cells were analyzed over unstained cells to show how positive gates for CD45, F4/80, CD11c and CD206 were located.

Supplementary Figure 4

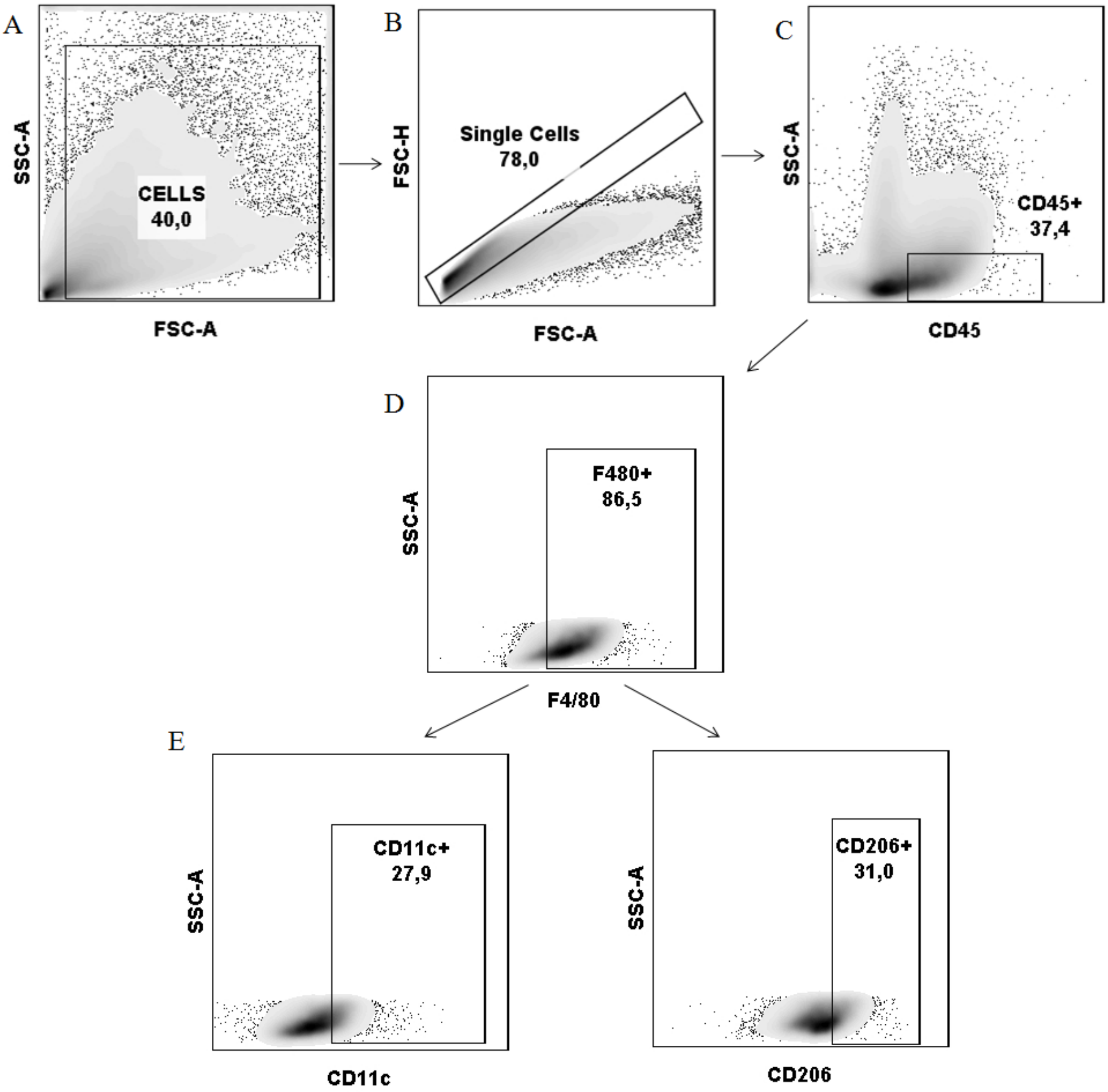

Supplementary Figure 4. Full gate strategy for analysis of resident wound macrophages. The lesions from WT or 5LO$^{-/-}$ mice, healthy or diabetic (T1D), were collected and processed for cell isolation. Debris, dead cells (A), and doublets (B) were gated out; CD45^{+} cells were gated, and F4/80^{+} were gated inside the CD45^{+} population. Then, CD11c^{+} or CD206^{+} were selected inside the CD45^{+}F4/80^{+} population.
